# Volumetric study reveals the relationship between outcome and early radiographic response during bevacizumab-containing chemoradiotherapy for unresectable glioblastoma

**DOI:** 10.1007/s11060-021-03812-9

**Published:** 2021-07-28

**Authors:** Kosuke Takigawa, Nobuhiro Hata, Yuhei Michiwaki, Akio Hiwatashi, Hajime Yonezawa, Daisuke Kuga, Ryusuke Hatae, Yuhei Sangatsuda, Yutaka Fujioka, Yusuke Funakoshi, Ryosuke Otsuji, Aki Sako, Osamu Togao, Takashi Yoshiura, Koji Yoshimoto, Masahiro Mizoguchi

**Affiliations:** 1 Department of Neurosurgery, Graduate School of Medical Sciences, Kyushu University, 3-1-1 Maidashi, Higashi-ku, Fukuoka, 812-8582 Japan; 2 Department of Neurosurgery, International University of Health and Welfare, Narita Hospital, Chiba, Japan; 3 Department of Clinical Radiology, Graduate School of Medical Sciences, Kyushu University, Fukuoka, Japan; 4 Department of Neurosurgery, Graduate School of Medical and Dental Sciences, Kagoshima University, Kagoshima, Japan; 5 Department of Radiology, Graduate School of Medical and Dental Sciences, Kagoshima University, Kagoshima, Japan

**Keywords:** Bevacizumab, Early response, Glioblastoma, RANO criteria, Volumetric study

## Abstract

**Purpose:**

Although we have shown the clinical benefit of bevacizumab (BEV) in the treatment of unresectable newly diagnosed glioblastomas (nd-GBM), the relationship between early radiographic response and survival outcome remains unclear. We performed a volumetric study of early radiographic responses in nd-GBM treated with BEV.

**Methods:**

Twenty-two patients with unresectable nd-GBM treated with BEV during concurrent temozolomide radiotherapy were analyzed. An experienced neuroradiologist interpreted early responses on fluid-attenuated inversion recovery (FLAIR) and gadolinium-enhanced T1-weighted images (GdT1WI). Volumetric changes were evaluated using diffusion-weighted imaging (DWI) and GdT1WI according to the Response Assessment in Neuro-Oncology (RANO) criteria. The results were categorized into improved (complete response [CR] or partial response [PR]) or non-improved (stable disease [SD] or progressive disease [PD]) groups; outcomes were compared using Kaplan–Meier analysis.

**Results:**

The volumetric GdT1WI improvement was a significant predictive factor for overall survival (OS) prolongation (*p* = 0.0093, median OS: 24.7 vs. 13.6 months); however, FLAIR and DWI images were not predictive. The threshold for the neuroradiologist’s interpretation of improvement in GdT1WI was nearly 20% of volume reduction, which was lesser than 50%, the definition of PR applied in the RANO criteria. However, even less stringent neuroradiologist interpretation could successfully predict OS prolongation (improved vs. non-improved: *p* = 0.0067, median OS: 17.6 vs. 8.3 months). Significant impact of OS on the early response in volumetric GdT1WI was observed within the cut-off range of 20–50% (20%, *p* = 0.0315; 30%, *p* = 0.087; 40%, *p* = 0.0456).

**Conclusions:**

Early response during BEV-containing chemoradiation can be a predictive indicator of patient outcome in unresectable nd-GBM.

## Introduction

Glioblastoma (GBM) is one of the most common malignant brain tumors and has a poor prognosis. The current standard treatment for newly diagnosed GBM (nd-GBM) is maximal safe removal with concurrent temozolomide and radiation (TMZ-RT), followed by maintenance TMZ with, if possible, tumor-treating fields [1]. Despite such multimodal treatment, the median overall survival (OS) remains less than 2 years.

In addition to these treatments, the FDA approved bevacizumab (BEV), an anti-VEGF antibody molecular-targeted drug that produces an indirect antitumor activity via inhibition of tumor angiogenesis [2], as treatment for recurrent GBM in 2009. Thereafter, two randomized trials, AVAglio and RTOG0825, were conducted to verify the efficacy of BEV for the treatment of nd-GBM, resulting in only progression-free survival (PFS) prolongation, but failed to impact OS [3, 4]. Accordingly, there is no robust evidence supporting the efficacy of BEV treatment for nd-GBM; however, BEV has been approved in Japan as an insurance-covered first-line drug for GBM concurrently with its second-line application, considering the benefit of maintaining patient performance status [5]. Thereafter, Japanese institutes, including ours, have launched several real-world studies, which indicate the clinical benefits of optional first-line BEV for patients with severe clinical conditions [1, 6–10]. Practically, we selected first-line BEV for patients with unresectable GBM and accumulated the clinical data [11]. These case series revealed that the radiographic course following first-line BEV for unresectable tumors varied among patients, and its outcome was considered an unresolved issue that needs to be addressed.

In the response judgment of GBM, gadolinium-enhanced T1-weighted image (GdT1WI) measurement based on the Macdonald criteria has generally been applied [12]. However, in BEV treatment for GBM, apparent tumor reduction on GdT1WI, the so-called pseudo-response, can be observed at an earlier time. Therefore, the evaluation of response using GdT1WI alone may overestimate the therapeutic effect of BEV. This type of complicated radiographic response during BEV treatment was taken into consideration, and the Response Assessment in Neuro-Oncology (RANO) criteria added the evaluation of non-enhanced lesions using T2/FLAIR [13]. Consequently, integrated evaluation based on multiple magnetic resonance imaging (MRI) sequences became essential for the assessment of treatment response; however, the association between complicated radiographic response and clinical outcome remains a controversial issue.

Only a limited number of studies have explored the relationship between radiographic response and clinical outcome following BEV treatment for nd-GBM [14, 15]. In this study, we retrospectively examined the detailed radiographic response using MRI scans during TMZ-RT combined with BEV for unresectable nd-GBM, and aimed to elucidate the relationship between early radiographic response and clinical outcome.

## Methods

### Patients

Since the Japanese approval of BEV for GBM in 2013, 72 adult (> 18 y) patients with IDH-wt nd-GBM were registered in our brain tumor database. Adaptive add-on BEV treatment to the Stupp regimen described previously [8–10] was selected for patients with unresectable GBM in our institute. The patients, whose postsurgical residual tumors were radiographically evident, were included in the present study. Two patients were excluded because BEV was added for the treatment of a novel lesion during radiotherapy, or radiographical total resection was performed previously. Finally, 22 patients were enrolled in the present study (Table 1). During radiotherapy, all enrolled patients underwent biweekly BEV administrations in combination with TMZ (mean 2.81 times; min 1; max 4). Steroid (betamethasone) was administered in three patients for neurological symptom control during concurrent chemoradiation therapy (CCRT). Two patients continued with 2 mg/day, whereas one patient started with 8 mg/day, which was gradually decreased to 2 mg/day. Thereafter, except for three patients who were transferred to the best supportive care and one who continued TMZ monotherapy, 18 patients continued maintenance treatment with BEV in combination with TMZ in accordance with the AVAglio regimen [3] (TMZ: mean 11.5 course [min 1; max 29], BEV: mean 19.1 course [min 2; max 36]). Although there is a heterogeneity in post-therapy, all 22 cases including these were used for the following contents. The present investigation was approved by the ethics committee (ethics review number: 2019-090) and was conducted in accordance with the 1964 Declaration of Helsinki (as revised in Fortaleza, Brazil, October 2013). All patients provided written informed consent.

**Table 1:** Clinical and molecular characteristics of patients

Characteristics	
Age, years (mean ± SD)	65.2 ± 12.0
Gender, n (%)	
Male	11/22(50.0)
Female	11/22(50.0)
KPS score, points (mean ± SD)	69.1 ± 21.1
*MGMT* status, n (%)	
Methylated	10/22(45.5)
Unmethylated	12/22(54.5)
*TERT* status, n (%)	
Mutant	13/22(59.1)
Wild-type	9/22(40.9)
*PTEN*, n (%)	
Heterozygous deletion	12/22(54.5)
Normal	10/22(45.5)
*EGFR*, n (%)	
Amplification	8/22(36.4)
Normal	14/22(63.6)
*CDKN2A*, n (%)	
Homozygous deletion	8/22(36.4)
Normal	14/22(63.6)

### Neuroimaging findings

**Fig. 1:**
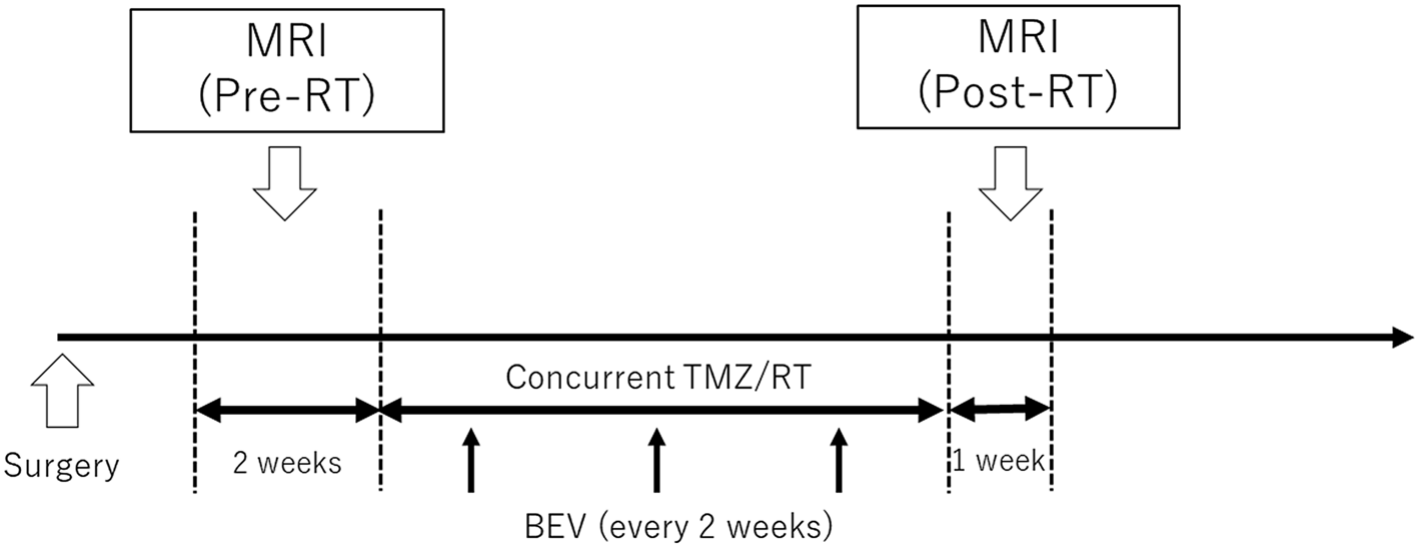
Schematic overview of the present study design

We evaluated the change in MRI images between pre- and post-CCRT (pre-RT and post-RT) (Fig. 1). Pre-RT MRI was obtained within 2 weeks prior to day 1 of CCRT, whereas post-RT MRI was performed within 1 week after CCRT. Post-MRI was performed within 1 week after RT. An experienced neuroradiologist (A.H.) who was blinded to patients’ clinical characteristics or prognosis evaluated the fluid-attenuated inversion recovery (FLAIR) and GdT1WI neuroimages. The neuroradiologist diagnosed the following based on pre-MRI and post-MRI GdT1WI (Gd-interpretation [Gd-IP]): (1) improved disease, defined as an obvious reduction in enhancing lesions; (2) progressive disease, defined as new or apparently enlarged enhancing lesions; and (3) stable disease, defined as neither improvement nor progression.

FLAIR was evaluated according to the RANO criteria methods [13]. Improvement in diffusion-weighted imaging (DWI) was evaluated using the measurement methods proposed by another group [16]. RANO criteria-based judgments on GdT1WI images that measured enhancing lesions and the sum of the product of the perpendicular diameter (Gd-SPPD) were also performed, and patients were categorized into two groups: improvement (complete response [CR] or partial response [PR]) and non-improvement (stable disease [SD] or progressive disease [PD]) [13].

### Genetic analyses

Sample preparations and subsequent confirmation of genetic signatures whose clinical significance was revealed in previous studies, including ours, were performed as described previously [17–25]. The status of *TP53*, *EGFR*, *PTEN*, and *CDKN2A* was evaluated using the MLPA kit P105-2 according to our previous study [26].

### Statistical analysis

Chi-squared and Fisher’s exact tests were used to investigate the relationship of neuroimaging changes with patient characteristics and molecular genetic stratification. Kaplan–Meier analysis was conducted to evaluate OS, and the log-rank test was used to compare survival distributions. The level of statistical significance was set at *p* < 0.05. All statistical analyses were performed using JMP Pro version 13 (SAS Institute Inc., NC, USA).

## Results

### Background characteristics

Patient characteristics and molecular genetic stratifications of tumors are summarized in Table 1. The judgment of improvement on DWI, FLAIR, and Gd-SPPD was determined in 8 (36.4%), 16 (72.7%), and 13 (59.1%) patients, respectively. Table 2 shows the genetic markers analyzed in this study. In the univariate analysis, unmethylated *MGMT* status and *CDKN2A* homozygous deletion were associated with a poor prognosis (unmethylated *MGMT* status: HR 2.54, 95% confidence intervals [CI] 0.86–7.5, *CDKN2A*: HR 2.49, 95% CI 0.86–7.27, *p* = 0.093). Prognostic significances of analyzed genetic markers were also evaluated in the multivariate analysis, and unmethylated *MGMT* was the only predictor of poor prognosis (HR 8.51, 95% CI 1.25–58.1, *p* = 0.0288). *CDKN2A* status and *EGFR* status showed a trend toward poor prognosis (*CDKN2A*, HR 2.73, 95% CI 0.88–8.42, *p* = 0.0806; *EGFR*, HR 6.56, 95% CI 0.75–2.45, *p* = 0.0649) (Table 2).

**Table 2:** Prognostic factors about the genetic markers

Genetic marker	Case (n = 22)	Univariate analysis		Multivariate analysis	
		HR (95% CI)	*p* value	HR (95% CI)	*p* value
Unmethylated *MGMT* status	12 (54.5%)	2.54(0.86–7.5)	0.093	8.51 (1.25–58.1)	0.029*
*TERT* mutation	12(57.1%)	1.02(0.35–2.95)	0.973	0.65(0.12–3.46)	0.610
*EGFR* amplification	8(36.4%)	1.48(0.52–4.24)	0.464	6.56(0.75–2.45)	0.065
*CDKN2A* homozygous deletion	8(36.4%)	2.49(0.86–7.27)	0.093	2.73(0.88–8.42)	0.081
*PTEN* loss	12 (54.5%)	0.91(0.33–2.54)	0.864	0.16 (0.02–1.26)	0.082

*HR* hazard ratio, *CI* confidence interval

*Indicates values that are statistically significant (*p* < 0.05)

No significant bias in clinical and genetic backgrounds was detected between patients whose radiographic findings improved with each of the three judgments (Table 3).

**Table 3:** Comparison of clinical and molecular characteristics between radiographical findings

MRI imaging	DWI		*p*	FLAIR		*p*	GD-SPPD		*p*
Image judgment	Imp (8)	Non imp (14)		Imp (16)	Non imp (6)		Imp (13)	Non imp (9)	
Age (median, year)	71.4 ± 11.2	61.7 ± 11.3	0.07	65.7 ± 11.5	64 ± 14.3	0.78	66 ± 12.4	64.1 ± 12.1	0.72
Sex, M/F	4/4	7/7	0.74	9/7	2/4	0.37	7/6	4/5	0.55
KPS change (non-det/det)^*1^	7/1	11/3	1	13/3	5/1	1	11/2	7/2	1
Eloquent/non eloquent	4/4	9/5	0.66	10/6	3/3	0.65	7/6	6/3	0.67
Pre Gd-SPPD high/low^*2^	3/5	8/6	0.66	9/7	2/4	0.64	7/6	4/5	1
*TERT* wt/mut	5/3	4/10	0.19	7/9	2/4	1	5/8	4/5	1
*MGMT*, u/m	3/5	9/5	0.38	9/7	3/3	1	6/7	6/3	0.41
*PTEN* n/hetero	3/5	7/7	0.67	7/9	3/3	1	6/7	4/5	1
*EGFR* n/amp	4/4	10/4	0.39	10/6	4/2	1	8/5	6/3	1
*CDKN2A* n/homo	7/1	7/7	0.17	11/5	3/3	0.62	9/4	5/4	0.66

*det* deterioration, *wt* wild type, *mut* mutant, *u* unmethylated, *m* methylated, *hetero* heterozygous deletion, *amp* amplification, *homo* homozygous deletion, *imp* improvement, *non imp* non improvement

*1 deterioration:≧20 KPS score reduction

*2 Set the median of Pre MRI Gd-SPPD as the border

### Survival outcome of radiographic findings

**Fig. 2:**
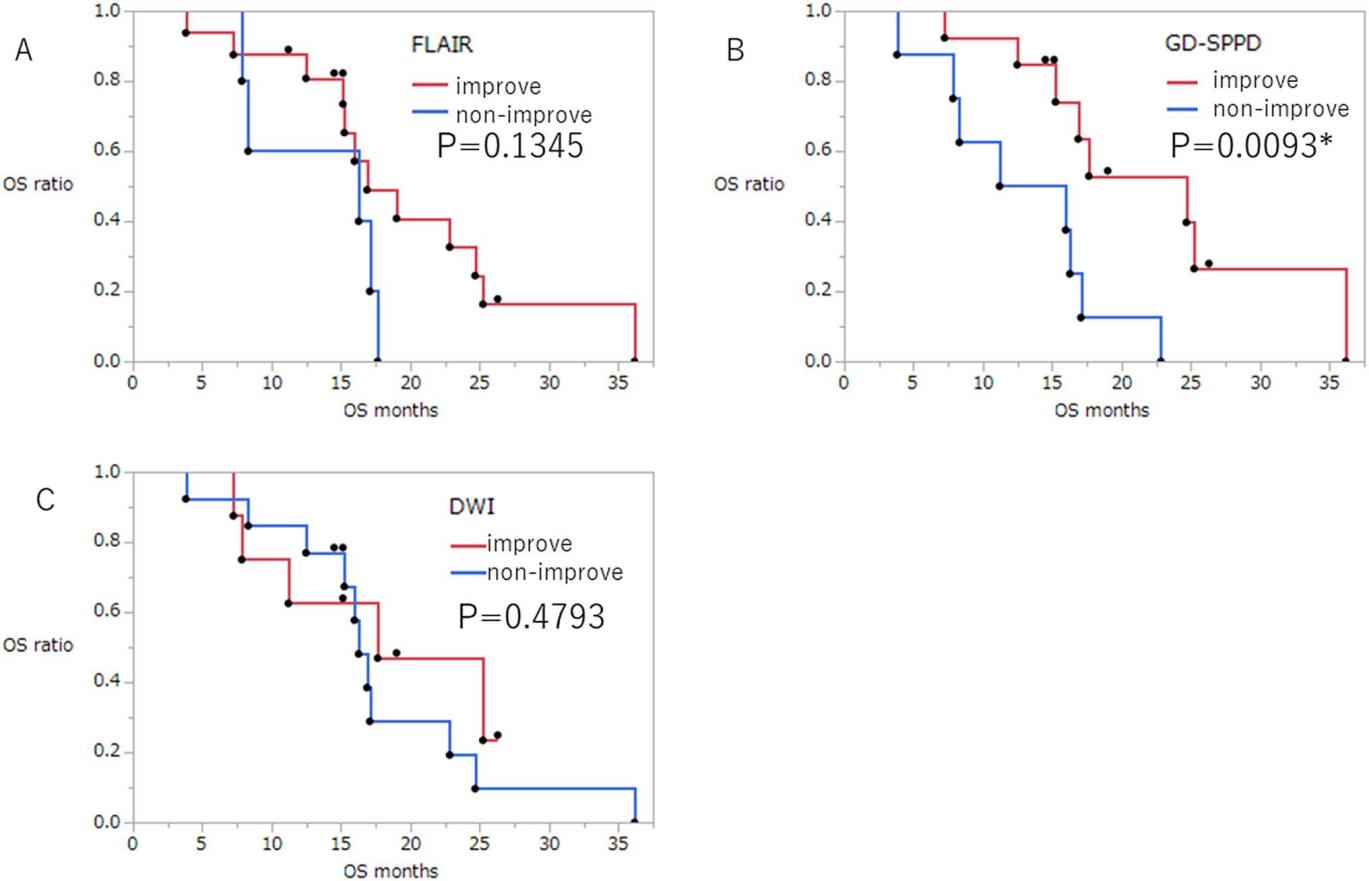
Kaplan–Meier estimates of overall survival (OS). **A** Fluid-attenuated inversion-recovery (FLAIR) imaging group stratified by improvement/non-improvement (SD or PD) do not show different outcomes. **B** Enhancing lesion and the sum of the product of the perpendicular diameter (Gd-SPPD) imaging group stratified by improvement (CR or PD)/non-improvement (SD or PD), and improvement is the significant predictive factor for OS prolongation (*p* = 0.0093). **C** diffusion-weighted imaging (DWI) group stratified by improvement (CR or PD)/non-improvement (SD or PD) do not show different outcomes. *CR* complete response; *OS* overall survival; *PD* progressive disease; *SD* stable disease

Kaplan–Meier analyses revealed that only Gd-SPPD improvement was a significant predictive factor for OS prolongation (*p* = 0.0093). The median OS was 24.7 and 13.6 months when GD-SPPD was improved or not, respectively. In contrast, FLAIR and DWI images were not predictive of OS outcome (FLAIR improved vs. non-improvement: *p* = 0.13, 16.9 vs. 16.3 months, DWI improved vs. non-improved: *p* = 0.48: 17.6 vs. 16.3 months) (Fig. 2).

**Fig. 3:**
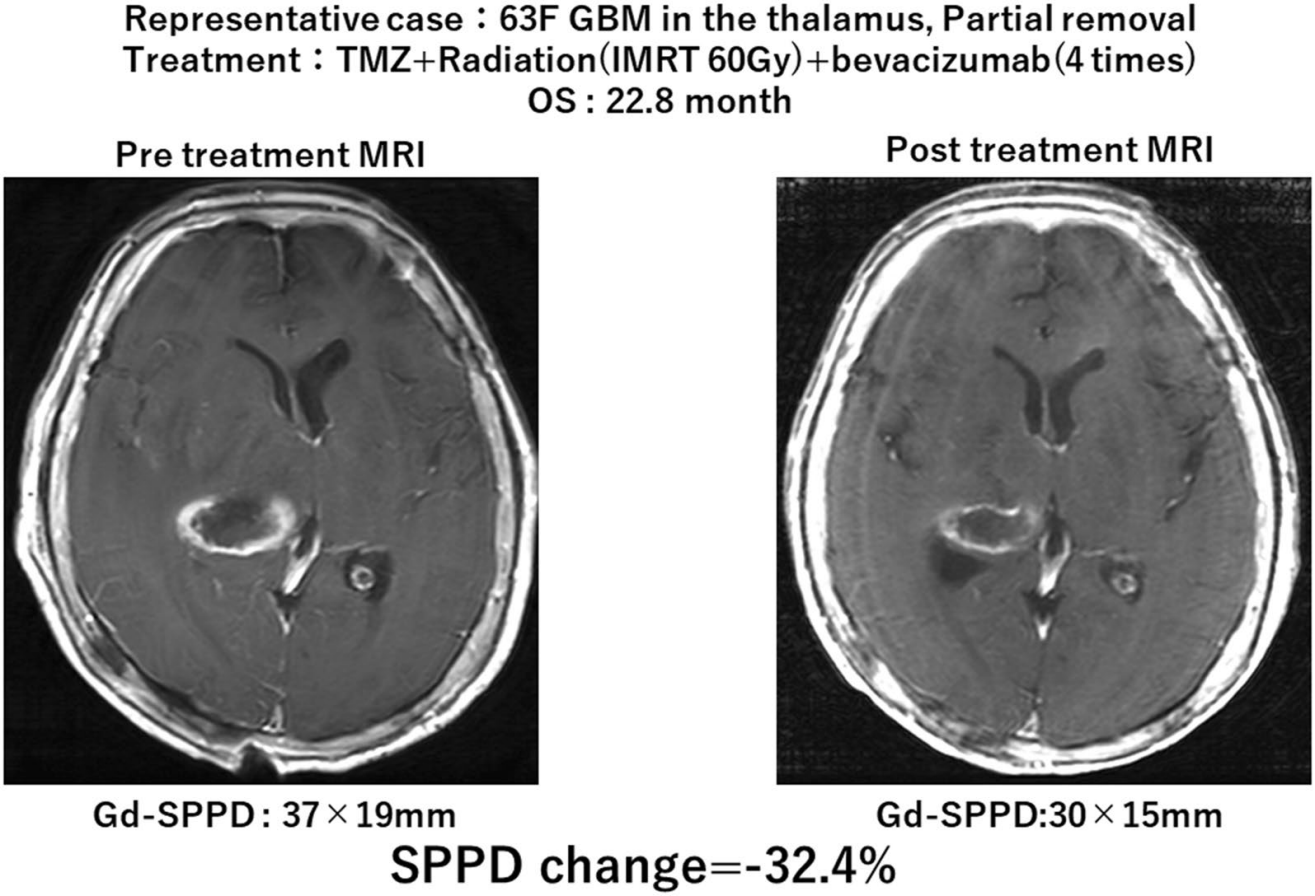
A representative case of discrepant result: 63-year-old female. We performed partial removal of the tumor. The patient was treated with temozolomide (TMZ; 75 mg/m^2^/day) and radiotherapy (Intensity Modulated RT60 Gy) and bevacizumab (four times in total). On the pre-RT magnetic resonance imaging (MRI), enhancing lesion and the sum of the product of the perpendicular diameter (Gd-SPPD) was 37 × 19 mm. After RT, GdT1WI showed a reduction in interpretation by the neuroradiologist. However, Gd-SPPD was 30 × 15 mm and the change in SPPD was 32.4%, which was determined as SD based on the Response Assessment in Neuro-Oncology (RANO) criteria. OS at 22.8 months. *GBM* glioblastoma; *RT* radiotherapy; *SD* stable disease; *TMZ* temozolomide

In addition to the evaluation of Gd-SPPD, GdT1WI improvement was interpreted by a neuroradiologist (Gd-IP). Improvement in Gd-IP was associated with OS prolongation (improvement vs. non-improvement: *p* = 0.0067, 17.6 vs. 8.3 months). We compared the discrepant judgment between Gd-SPPD and Gd-IP results for seven cases in which the reduction was less than 50% in the measurement, as shown in Fig. 3 (Table 4).

**Table 4:** Cases that were judged SD in the Gd-SPPD due to reduction of less than 50%

Case	Gd-interpretation	Gd-SPPD (%)	Gd-SPPD judge	Discrepant of judgement
1	Imp	− 40.4	SD	Yes
2	Imp	− 32.4	SD	Yes
3	Imp	− 28.8	SD	Yes
4	Non imp	− 11.1	SD	No
5	Non imp	− 8.68	SD	No
6	Non imp	− 8.03	SD	No
7	Non imp	− 7.53	SD	No

*SD* stable disease, *imp* improvement, *non imp* non improvement

**Fig. 4:**
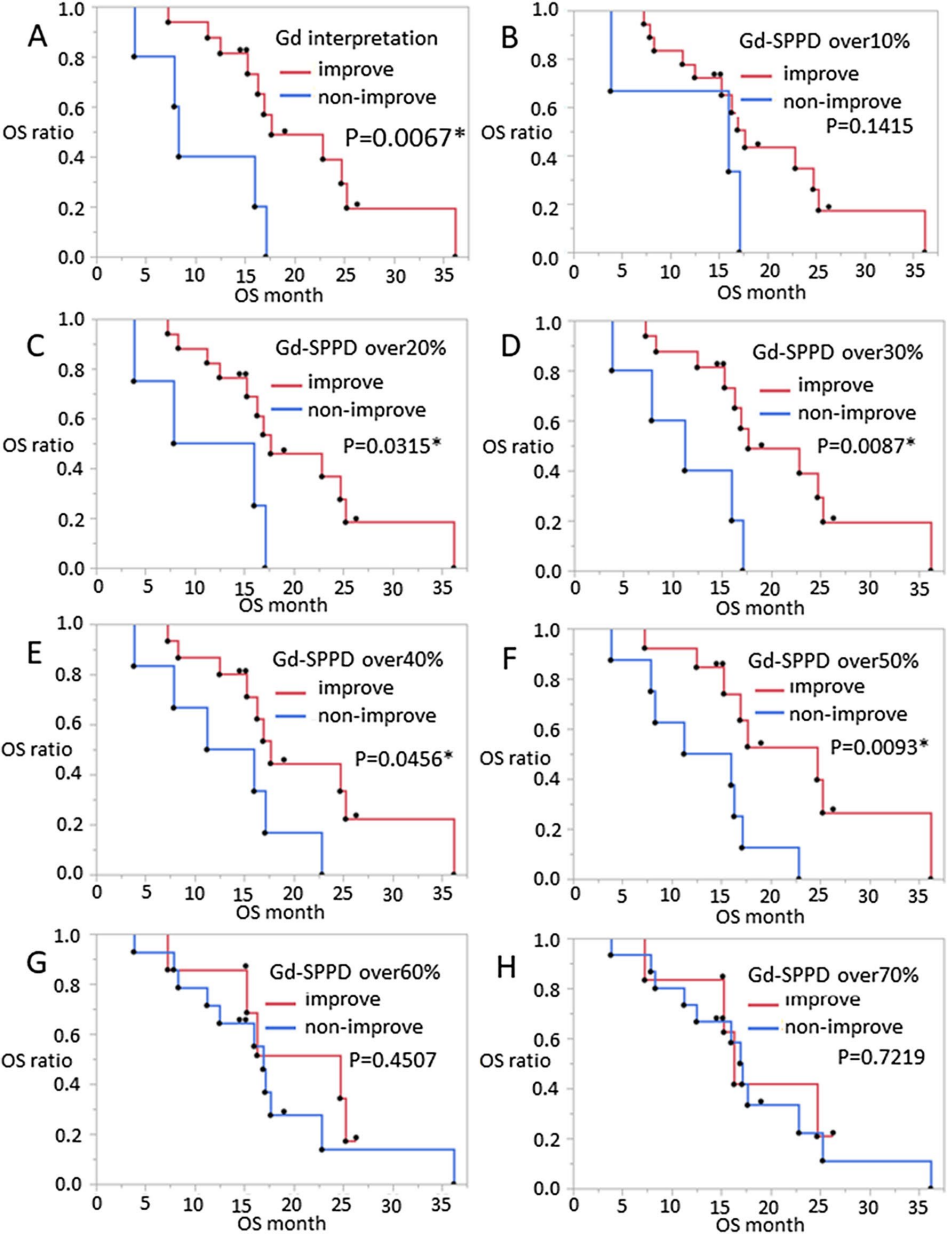
Correlation between Gd-improvement and outcome. **A** Significant overall OS prolongation is revealed in the Gd-interpretation improvement group. **C**–**F** OS prolongation is observed in the improvement group according to a cut-off line from 20 to 50% (20% improved vs. non-improved: *p* = 0.0315, 30% improved vs. non-improved: *p* = 0.087, 40% improved vs. non improved: *p* = 0.0456). **B**, **G**, **H** On the other hand, this outcome disappeared when using a 10%, 60%, and 70% cut-off line. *OS* overall survival; *PR* partial response

To determine the suitable threshold of GdT1 improvement for the prediction of outcome, we changed the cut-off line for PR judgment in the measurement from 10 to 70%, and we performed Kaplan–Meier analysis (Fig. 4). OS prolongation was observed as a cut-off of 20% to 50% (20% improvement vs. non-improvement: *p* = 0.0315, 30% improvement vs. non-improvement: *p* = 0.087, 40% improvement vs. non-improvement: *p* = 0.0456).

### BEV toxicity

During the course of CCRT, the only obvious BEV-related toxicity was deep venous thrombosis, which was identified in a single patient and led to a temporary discontinuation of BEV administration.

## Discussion

We analyzed the impact of radiographic changes during BEV-containing chemoradiotherapy for unresectable nd-GBM. As a result, while changes in DWI and FLAIR images did not have a significant impact, only GdT1WI improvement was associated with significant OS prolongation. The Macdonald criteria applied to GdT1WI have been the standard for determining the treatment response of GBM. The RANO criteria added FLAIR image progression to predict pseudo-response [12, 13]. In previous reports, the relationship between the radiographic response assessed by the RANO criteria and survival outcome following BEV treatment was analyzed in patients with recurrent GBM. Ellingson et al. reported that the changes in FLAIR and GdT1WI were not related to both PFS and OS, and the pre-treatment ratio of FLAIR to contrast-enhancing volume was a predictive marker of both PFS and OS [27]. Boxerman et al. reported that early progression of GdT1WI was a poor prognostic factor for OS and that changes in FLAIR images showed no significant impact on OS [28]. Both studies investigated recurrent GBM, and the therapeutic situations were different from those for nd-GBM. These two studies indicated that quantitative FLAIR improvement showed no significant correlation with OS because BEV treatment improved FLAIR hyperintensity with an anti-permeability effect and supported our results that an early response on FLAIR images is not likely to reflect the survival outcome after BEV treatment. It is speculated that FLAIR progression is useful for differentiating the pseudo response and that FLAIR improvement does not indicate an antitumor effect. An exploratory analysis of AVAglio classified the type of radiologic progression of nd-GBM treated with TMZ-RT and BEV, revealing that CR in the GdT1WI group showed longer OS than that in the PR group [12]. Our results indicated that even PR on GDT1WI could have a survival impact in real-world clinics. The discrepancy between AVAglio and our results might be due to differences in the background characteristics. Our case series consisted of patients with severe clinical conditions that were more likely to be excluded from clinical trials due to their strict inclusion criteria. In addition, the extent of resection should be taken into consideration because CR on GdT1WI is likely to occur after treatment of patients with near completely resected tumors, while there were very few such cases included in our cohort. The impact of GdT1WI improvement in clinical practice is currently an unsolved issue to be elucidated by the accumulation of clinical reports from Japanese institutes where BEV is approved for treatment of nd-GBM.

Other MRI sequences were analyzed for their impact on outcome. DWI has been recognized as a promising sequence for the prediction of the response to BEV treatment because the apparent diffusion coefficient level can reflect the cellularity of tumor tissues [29]. Yamasaki et al. reported that DWI can distinguish the pseudo-response from true response after BEV treatment for recurrent GBM, and the evaluation based on the RANO criteria predicted OS more precisely when combined with DWI change, suggesting that DWI can clearly demonstrate the true extent of the tumor area at an early point [12]. In this study, DWI evaluation was performed according to the method of Yamasaki et al.; however, there was no correlation between the OS and DWI responses. It is noteworthy that the impact of radiotherapy should be considered when discussing these issues because the treatment situation is different between nd- and recurrent GBM. Regarding nd-GBM, relative cerebral blood volume (rCBV) is attracting attention for its association with the response to BEV treatment. An exploratory analysis of RTOG0825 revealed OS prolongation with BEV treatment in the high pretreatment rCBV group compared to the placebo group [30]. However, rCBV changes during BEV treatment did not show an impact on OS in both nd- and recurrent GBMs [30, 31]. Another recent study focused on the contrast between DWI and perfusion images to generate an automated threshold by measuring the hypercellular tumor volume and hyperperfused tumor volume and showed that the ratio changes of these two values during chemoradiotherapy had an impact on OS [32]. On the other hand, they reported that significant GdT1WI volume reduction during chemoradiotherapy was also observed; however, the GdT1WI volume change was not correlated with OS. Nonetheless, the treatment situation in this study was also different from that in ours, in which chemoradiation included BEV administration. Further studies including multiple MRI sequences are warranted to confirm the relationship between early radiographic response and outcome in clinical situations where first-line BEV is approved.

Our study revealed that Gd-improvement evaluated not only by the RANO criteria but also by neuroradiologist’s impression can predict the outcome of unresectable GBM treated with BEV-combined regimen. In addition, the extent of GD-SPPD improvement correlated with significant outcome impact ranging from 20 to 50%, similar to the GD-IP and GD-SPPD thresholds. In other words, OS prolongation can be predicted even in cases when GD-improvement is insufficient to determine PR according to the RANO criteria. These results indicated that, for outcome prediction, evaluation by a neuroradiologist hinted at a clinically appropriate response judgment of BEV-combined treatment for nd-GBM. The criteria of radiographic response by measuring contrast-enhancement lesions have been consistently used from the Macdonald criteria to the RANO criteria [12, 13]. McDonald's standard was created based on the WHO oncology response criteria, which is a general diagnostic imaging standard for solid tumors [33]; therefore, the measurement protocol for contrast-enhancement lesions has not been changed for more than 20 years. Our results propose the possibility that some patients evaluated as SD by the RANO criteria may have a good prognosis and suggest an alternative threshold value for identifying the group with a good prognosis [26].

Our study has several limitations. First, this was a single-center, non-randomized, retrospective study that included a small number of patients. Hence, our results should be verified in a larger cohort. As first-line BEV for GBM has not been approved outside of Japan, a multi-institutional clinical study involving several Japanese facilities is desirable. Second, subsequent treatments were inconsistent among the enrolled patients, which might have affected the outcome. Third, the analyzed image sequences were limited to only DWI, Gd, and FLAIR, and other sequences such as rCBV may be more significant predictors. Fourth, while there existed a correlation between the extent of GD-improvement and outcome, how the background bioactivity attributed to such a relationship was unclear. In the present study, univariate analysis for molecular markers revealed an unmethylated *MGMT* status, and *CDKN2A* homozygous deletion showed a trend toward poor prognosis. Our recent study also reported that MGMT and CDKN2A status could stratify Japanese GBM patients into three race-specific groups with different prognoses [26]. Further accumulation of studies including molecular-genetic signatures and evidence beyond real-clinic data are warranted to evaluate the significance of image changes during BEV-included regimens for unresectable GBM.

## Conclusions

We examined the radiographic response in multiple MRI sequences (FLAIR, Gd-SPPD, and DWI) in patients with unresectable nd-GBM treated with BEV-including chemoradiotherapy and proved that the Gd-SPPD improvement group showed a significant prolongation of OS. Furthermore, the OS impact was significant even with less strict judgment of radiographic response compared with that of the RANO criteria. This raised the possibility that some patients evaluated as SD by the RANO criteria may have a good prognosis, and the interpretation of neuroradiologists likely hinted at an alternative evaluation for outcome prediction.
